# Modeling and probabilistic analysis of civil aircraft operational risk for suborbital disintegration accidents

**DOI:** 10.1371/journal.pone.0266514

**Published:** 2022-04-07

**Authors:** Wantong Chen, Shuyu Tian, Shiyu Ren

**Affiliations:** Department of Electronic Information and Automation, Civil Aviation University of China, Tianjin, China; Southeast University, CHINA

## Abstract

To reduce the collision risk to civil airliners caused by suborbital vehicle disintegration events, this paper uses a covariance propagation algorithm to model the debris landing point of suborbital disintegration accidents and gives a collision probability analysis method for civil airliners encountering debris during the cruise. Collision warning is performed for airborne risk targets to improve the emergency response capability of the ATC surveillance system to hazardous situations. The algorithm models the three-dimensional spatial motion target localization problem as a Gauss-Markov process, quantifying the location of debris landing points in the vicinity of nominal trajectories. By predicting the aircraft trajectory, the calculation of the inter-target collision probability is converted into an integration problem of a two-dimensional normally distributed probability density function in a circular domain. Compared with the traditional Monte Carlo method, the calculation speed of debris drop points is improved, which can meet the requirements of civil aviation for real-time response to unexpected situations.

## 1. Introduction

In recent years, along with the shift from government-driven to market-driven drivers of space activities, commercial spaceflight has become an important driver of global space activities, and the commercialization of suborbital flight has developed rapidly [1]. Unlike civil aviation flight methods, the suborbital flight is a flight between both gravity and aerodynamics, as the speed of flight around the earth is not reached, the vehicle is equivalent to making a parabola back to earth in space [2]. Compared to a normal flight, the suborbital flight time will be shortened several times; compared to a spacecraft, suborbital vehicles are cheap and reusable, thus presenting a huge economic value [3]. It is predicted that suborbital transportation is likely to become one of the main modes of space travel in the next generation [4].

Suborbital vehicles travel at hypersonic speeds, with orbits between the maximum altitude of existing aircraft and the minimum orbital altitude of satellites, and are prone to disintegration and large debris generation under strong aerodynamic loads. The Inter-Agency Space Debris Coordination Committee (IADC) Space Debris Mitigation Guidelines [5] state that spacecraft typically disintegrate at an altitude of 75 km, and that a typical spacecraft disintegration altitude of (80±10) km has been observed in practice, with object re-entry survival The aerodynamic forces on these fragments in the atmosphere are somewhat random and their distribution and landing points are difficult to predict, with serious consequences in the event of a collision with a civil aircraft [6]. During space launches, traffic control authorities completely close off large, infinitely high areas of "hazardous airspace [7]" in advance of the launch plan. This measure avoids aircraft encounters with risky objects and maximizes the safety of civil aviation operations, but also causes extensive flight delays and economic losses. As the number of commercial suborbital space activities increases in the future, this static, excessive, and prolonged approach to traditional airspace management will be costly to airlines [8].

Suborbital debris distribution modeling is a key technique to form the danger zone prediction, and effective danger zone prediction is a prerequisite for implementing safe air traffic control. In terms of debris distribution modeling, some scholars have approximated the suborbital debris prediction hazard zone as a rectangle based on the debris statistics of the Columbia disintegration event [9–11], with the length of the rectangle being the disintegration height divided by 1000 and the width of the rectangle being 1/8 of the length; this approximation is relatively crude, lacks rigorous scientific justification, and does not have universal significance. Some scholars have used the TAP (Trajectory Analysis Program) model to simulate the debris distribution in a visual simulation system for suborbital accident debris risk assessment [12, 13], which was first designed to simulate the debris distribution generated by the accidental disintegration of an aircraft in an aerobatic stunt show and is not essentially applicable to the trajectory prediction of suborbital disintegration accident debris. In addition, in risky target collision warnings, some scholars use the orbital state and error covariance information obtained from the forecast to calculate the collision probability between targets, but this algorithm is only applicable to outer space targets.

To improve the real-time response capability of ATMS to suborbital disintegration accidents and minimize the waste of airspace resources, this paper uses a covariance propagation algorithm to model the suborbital disintegration accident debris position distribution as a Gauss-Markov process, combined with the classical aircraft track prediction model, fully considering the safety requirements in the civil aviation field and the response speed requirements of ATC system, the collision between aircraft and suborbital debris The probability is calculated, and the calculation results can provide real-time reference information for air traffic controllers to issue redirect instructions to pilots for air risk target collision warning.

## 2. Collision model

### 2.1. Covariance propagation method for debris model

Reyhanoglu *et al*. [14] proposed a covariance propagation algorithm for determining the four-dimensional footprint of debris based on the three-degree-of-freedom (3-DOF) model of falling objects on a rotating planet. The algorithm is based on aerodynamics and Newton’s laws of motion to establish a mathematical model of the diffusive motion of individual debris in a complex atmospheric environment, and further analysis is performed. Let *x*_1_*x*_2_*x*_3_ denote the position of the debris in the station center coordinate system at the moment of aircraft disintegration. The origin of the coordinate system is at (*θ*_0_, *ϕ*_0_) the surface of the earth, where (*θ*_0_, *ϕ*_0_) is the initial latitude and longitude at the moment of aircraft disintegration. The *x*_1_ axis points to the east and the *x*_2_ axis points to the north, the *x*_1_*x*_2_ plane represents the plane tangent to the earth at the origin, and the *x*_3_ axis is perpendicular to the plane pointing to the zenith direction. The coordinate system can also be called the ENU (East-North-Up) coordinate system. Then the equation of motion describing the propagation trajectory of the debris can be given by the following equation:

$$\begin{array}{l} \dot{x}=v\\ \dot{v}=a_{D}+a_{G}-2\omega \times v-\omega \times \text{[}\omega \times (x+R_{e}{\hat{e}}_{3})]+\xi \end{array}$$ (1)

Where: ***x*** = [*x*_1_, *x*_2_, *x*_3_]^*T*^ and ***v*** = [*v*_1_, *v*_2_, *v*_3_]^*T*^ represent the position vector and velocity vector in the ENU coordinate system, respectively; ***ω*** = [0,*ω*_*e*_ cos *ϕ*_0_, *ω*_*e*_ sin *ϕ*_0_]^*T*^ is the rotational angular velocity vector in the ENU coordinate system, where *ω*_*e*_ = 7.2921 × 10^−5^ rad/s represents the mean angular velocity of the Earth’s rotation; ***e***_3_ = [0, 0, 1]^*T*^ represents the third standard unit vector; *ξ* represents the random acceleration vector caused by modeling uncertainty and disturbances; *R*_*e*_ = 6378 × 10^3^ m represents the radius of the Earth, and according to the inverse square gravity model, the gravitational acceleration can be represented by *g* = *g*_*e*_(*R*_*e*_/(*R*_*e*_ + *x*_3_))^2^, where *g*_*e*_ = 9.81/s^2^; ***a***_*D*_ represents the instantaneous acceleration associated with the atmospheric density, which can be expressed as $a_{D}(t)=-\frac{1}{2}\frac{\rho \left|v_{a}(t)\right|v_{a}(t)}{\beta }$, where *β* represents the ballistic coefficient, and ***v***_*a*_ is the difference between the velocity vector ***v*** of the debris and the wind vector ***w***(***x***,*t*), ***v***_*a*_ = ***v*** − ***w***(***x***, *t*).***a***_*G*_ represents the acceleration of gravity, $a_{G}=g{\hat{e}}_{3}$.The atmospheric index model can be expressed as $\rho (x_{3}\text{)=}\rho_{e}\;\text{exp}\left(\frac{-x_{3}}{H}\right)$.

For debris generated from suborbital disintegration accidents, the Monte Carlo method is usually used to simulate the Spatio-temporal evolution process of their state vectors. However, in practical applications, the simulation process of a large amount of debris is time-consuming and cannot meet the high requirements of real-time response for air traffic control work. Therefore, the estimation of debris drop-off points using the Covariance Analysis Description Equation Technique (CADET) can significantly reduce the computational effort. The algorithm describes the stochastic process of predicting debris drop points around a linearized nominal trajectory as a Gauss-Markov process and then constructs a probability ellipsoid of the Gauss-Markov process at a certain confidence level through a probability density function to quantify the location of debris drop points near the nominal trajectory.

First, define the extended state vector ***s*** = [***x***^*T*^, ***v***^*T*^]^*T*^, then the fragment motion Eq (1) can be reformulated as:

$$\dot{s}=f(s)+\eta$$ (2)

Where: ***η*** = [***0***^T^, ***ξ***^T^]^T^. Define ${\dot{s}}_{*}$ as the nominal state vector, i.e., ${\dot{s}}_{*}=f(s_{*})$, and the perturbation vector **ɀ** = ***s***–s_*_, then Eq (2) is linearized as:

$$\dot{z}=A(t)z+B\xi (t)$$ (3)

Where: $A\left(t\right)=\frac{\partial f}{\partial s}\left(s_{*}\right)$, $B=\left[\frac{0_{3\times 3}}{I_{3\times 3}}\right]$. Assuming that the initial state obeys the Gaussian distribution and that the noise statistics are characterized by $E\left[\xi \left(t\right)\right]=\bar{\xi }\left(t\right)$ and $E\left[\left(\xi \left(t\right)-\bar{\xi }\left(t\right)\right){\left(\xi \left(t\right)-\bar{\xi }\left(t\right)\right)}^{T}\right]=\Xi \left(t\right)$, then Eq (3) can be regarded as a continuous Gauss-Markov process. The fragment position vector at a time *t* can be expressed as a Gaussian random variable with mean $x\left(t\right)=C\left(\bar{z}\left(t\right)+s_{n}\left(t\right)\right)$ and covariance matrix ***X***(*t*) = *C****Z***(*t*)*C*^*T*^, where ***C*** = [***I***_3×3_ ***0***_3×3_].

**Fig 1:**
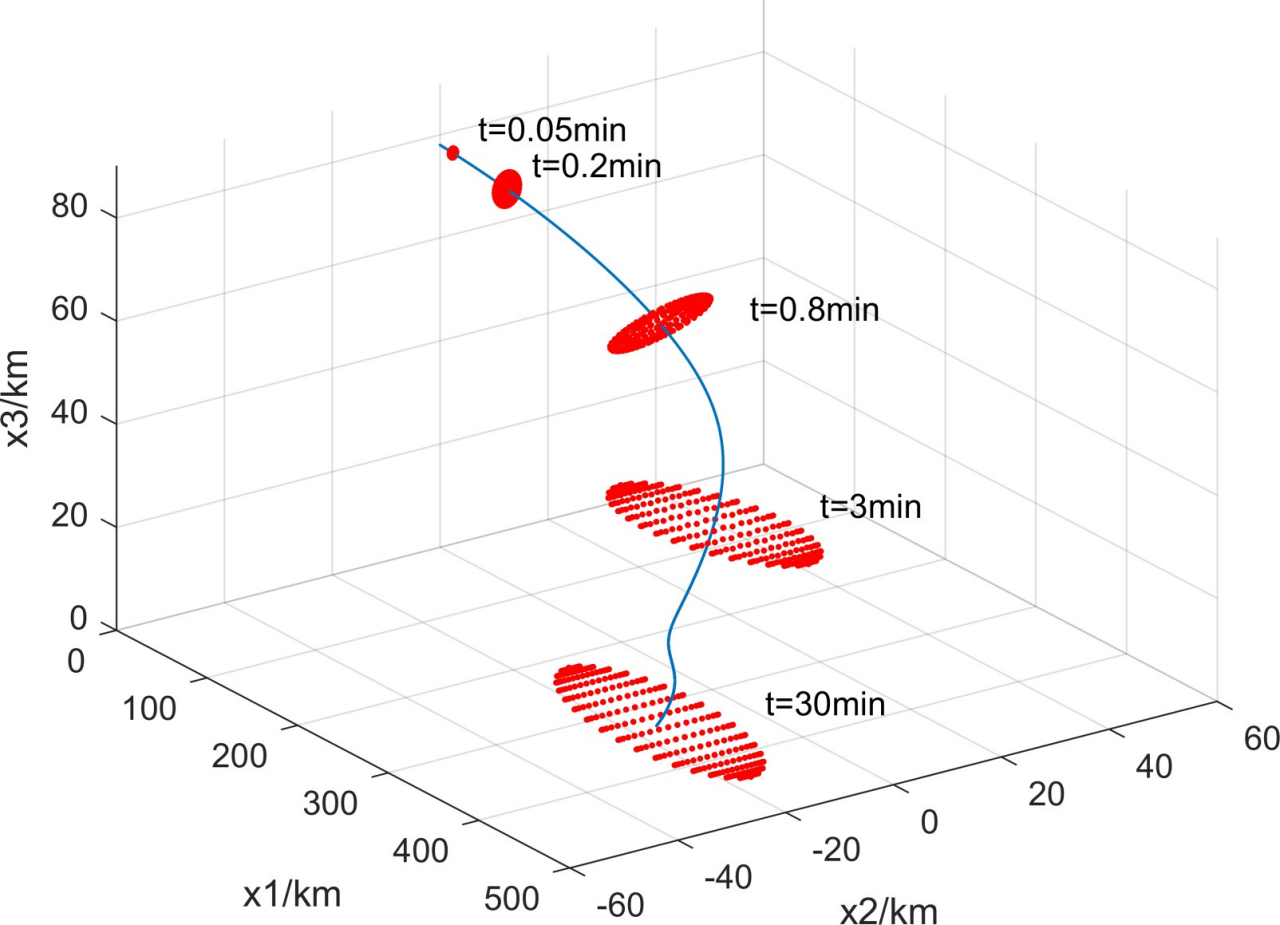
4-D footprint of debris.

In the above model, the main uncertainty influences include ballistic coefficients, atmospheric density, and wind vectors. The widely used empirical density model MSISE-00 (Mass Spectrometer Incoherent Scatter Radar Extended 2000 [15]) and the wind model HWM-07 (Horizontal Wind Model 2007 [16]) were chosen for modeling the disintegration accident. The debris ballistic coefficients can be characterized by probabilistic statistics. The motion state (longitude, latitude, altitude, velocity vector, heading angle, etc.) of the suborbital vehicle before disintegration can usually be derived from radar observations or flight trajectory equations. The description of the debris dispersion characteristics is extended to four dimensions by introducing an ellipsoidal set constructed by parameterizing the center and shape matrices concerning time. The resulting 4D probability constrained problem is much more complex than the 3D problem due to the infinite nature of the time variables. Therefore, solving the problem requires discretizing the time and associating each sample time with an ellipsoid. In this case, the 4D footprint of a single momentary fragment is part of the probability ellipsoid, while a series of instantaneous probability ellipsoids form a "fragment channel", as in Fig 1.

### 2.2. Aircraft track prediction model

**Fig 2:**
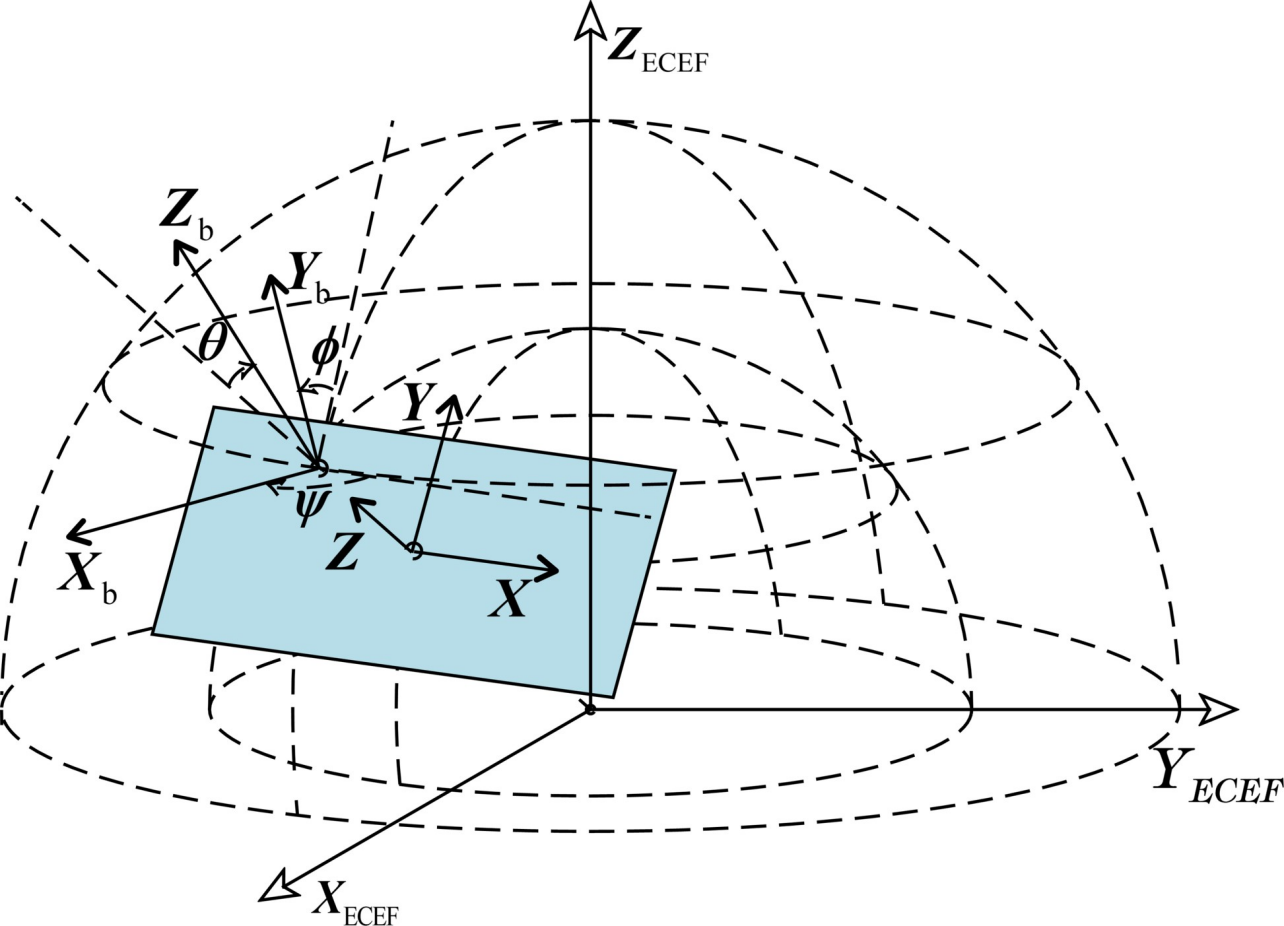
The definition of carrier coordinate system and navigation coordinate system.

The coordinate system describing the position of the moving target mainly includes the carrier coordinate system and the navigation coordinate system, and the target attitude is described by the pitch angle *θ*, roll angle *ϕ*, and yaw angle *ѱ*. The carrier coordinate system is the Front-Left-Up (FLU) reference system, and the navigation coordinate system is the ENU reference system, as shown in Fig 2, and the angular conversion relationship between the carrier coordinate system and the navigation coordinate system is represented by the Earth Centered Earth Fixed (ECEF) reference system.

**Fig 3:**
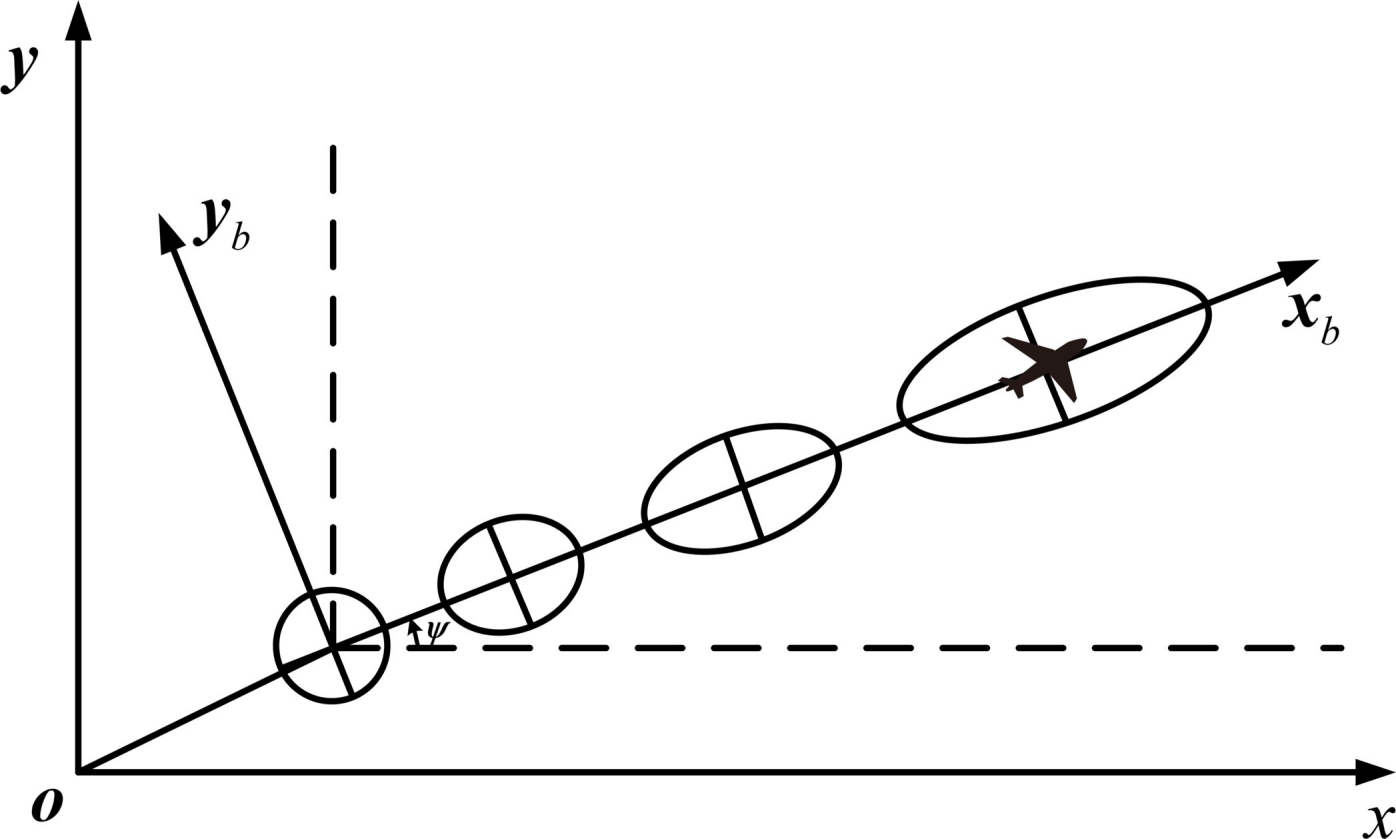
Trajectory error elliptical evolution in the carrier coordinate system.


In this paper, we only discuss the case that the aircraft encounters suborbital debris during the cruise (the cruise altitude of civil aircraft is generally around 10km), i.e., the pitch angle is considered to be 0 and the flight attitude is neglected. As shown in Fig 3, let be the angle between the aircraft heading and due east direction, and let **$R_{b}^{n}$** denote the coordinate conversion matrix from the carrier coordinate system to the navigation coordinate system.

$$R_{b}^{n}=\left[\begin{array}{ccc} \text{cos}\;\psi & -\text{sin}\;\psi & 0\\ \text{sin}\;\psi & \text{cos}\;\psi & 0\\ 0 & 0 & 1 \end{array}\right]$$ (4)

Due to the presence of uncertainties such as wind speed, navigation, and aircraft positioning errors, after studying and analyzing a large amount of track data, it is generally assumed that the track prediction error obeys normal distribution. Paielli et al. [17] proposed that the position error between the aircraft’s actual position within the horizontal altitude layer and the planned track within the next 30 minutes follows a zero-mean normal distribution and that the position prediction errors are independent of each other along the track direction and in the vertical track direction. In an aircraft equipped with Flight Management System (FMS), the aircraft adjusts the navigation position through a feedback mechanism. In the vertical track direction, the position closed-loop feedback can stabilize the Root Mean Square (RMS) error at a constant level, while in the along-track direction, due to the unpredictable wind direction, the FMS can only achieve feedback through airspeed, resulting in a classical linear growth of the along-track RMS error [18], and the along-track error and the vertical track error are independent of each other of the aircraft, the aircraft track error can be obtained as an ellipse with its long axis along the flight direction and its short axis perpendicular to the flight direction, as shown in Fig 3.

The position prediction error of the aircraft in the carrier coordinate system is a diagonal matrix. Suppose the aircraft position in the carrier coordinate system is ***q***, and the corresponding predicted position is $\bar{q}$, and the trajectory prediction error $\tilde{q}=q-\bar{q}$. The track prediction error approximately conforms to the normal distribution, and its covariance matrix is the diagonal matrix ***S***.

$$S=\left[\begin{array}{ccc} \sigma_{x}^{2} & 0 & 0\\ 0 & \sigma_{y}^{2} & 0\\ 0 & 0 & \sigma_{z}^{2} \end{array}\right]$$ (5)

Where: **σ**_*x*_ is the error along the heading direction, when the aircraft travels at a uniform speed in a straight line, ***σ***_*x*_ increases linearly with time, and satisfies:

$$\sigma_{x}=r_{a}\cdot t$$ (6)

Where: *r*_*a*_ = 0.25n.mi/min is the growth rate of error along with the heading.***σ***_*y*_ is the error along the vertical heading direction and satisfies:

$$\sigma_{y}=\text{min}\left\{r_{c}s(t),\;\text{ln}.\text{mi}\right\}$$ (7)

Where: *r*_*c*_ = 1/57 represents the error growth factor and *s*(*t*) represents the distance flown by aircraft in the predicted time. ***σ***_*z*_ is the error along the vertical heading direction and is usually assumed to be ***σ***_*z*_ = 30 m.

## 3. Collision probability calculation method

### 3.1. Definition of the encounter coordinate system

To avoid the impact of vehicle debris in suborbital disintegration accidents on the safe operation of civil airliners, the collision probability of debris and aircraft in the same space needs to be considered comprehensively for airborne risk target collision warning. To simplify the calculation process of collision probability, the calculation is generally based on the following assumptions [19]: the position vector and velocity vector of two targets in the navigation coordinate system during the encounter are known and can be equivalently transformed into a sphere of known radius; the motion process of two targets during the encounter has the characteristics of uniform linearity and no uncertainty in velocity, i.e., the position error ellipsoid is guaranteed to remain constant during the encounter; the position error of two targets obeys the normal distribution in three-dimensional space, which can be described by the predicted position and position error covariance matrix. The collision condition is that the distance between the two targets is less than the sum of their equivalent radii, and thus the collision probability is defined as the probability that the mode of the relative position vectors is less than the sum of their equivalent radii.

**Fig 4:**
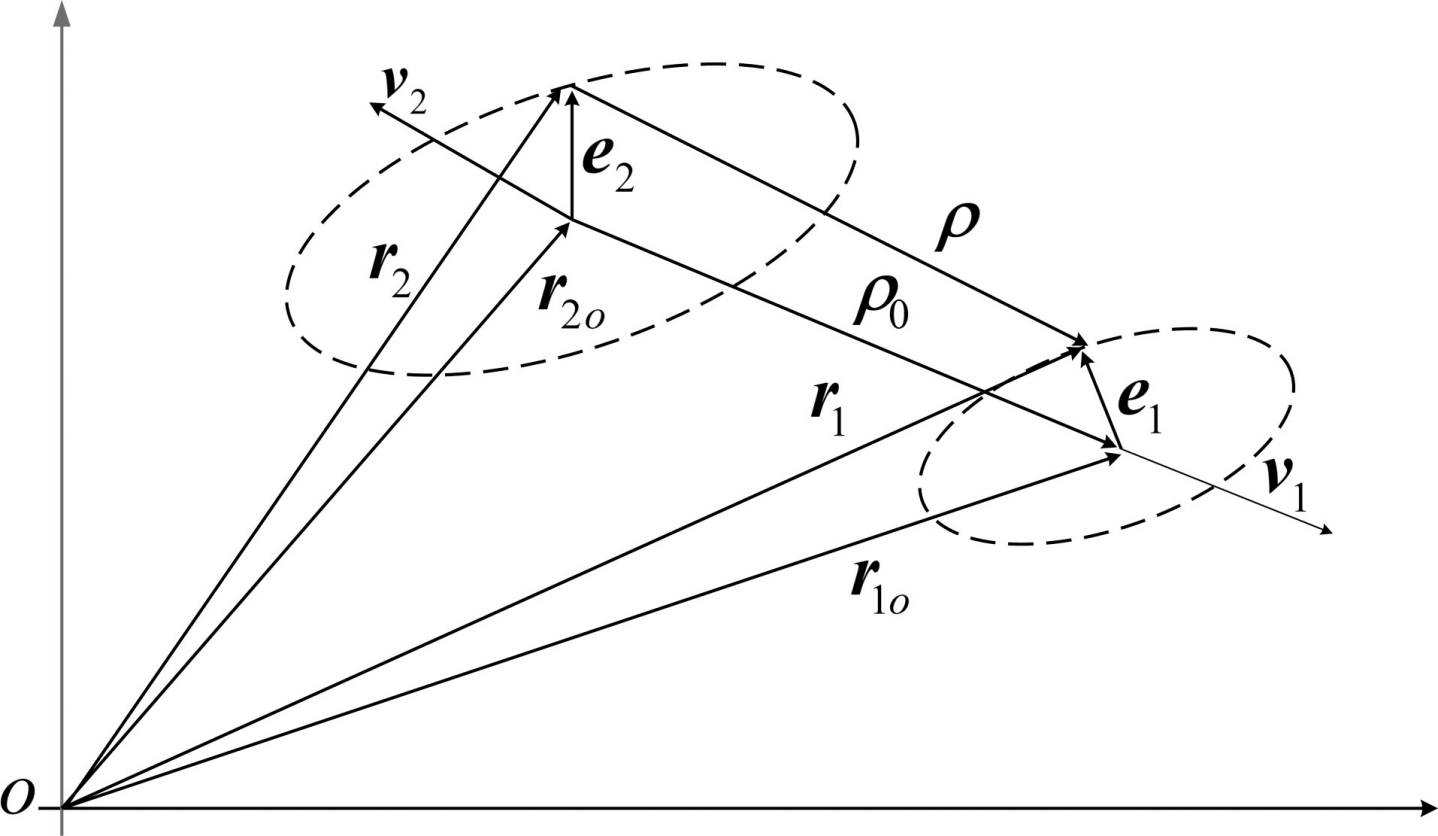
Geometrical relationship of position and velocity at encounter.

The position-velocity geometric relationship between the aircraft and the individual debris after a suborbital disintegration accident is shown in Fig 4. Where ***v***_1_, ***v***_2_ denotes the velocity vectors of the two targets, respectively, ***r***_1*o*_, ***r***_2*o*_ is the predicted position vector, and ***r***_1_, ***r***_2_ is the actual position vector. The position errors of the two targets are expressed in the carrier coordinate system as ***e***_1_, ***e***_2_, and the equivalent radii are *R*_1_, *R*_2_, respectively. The actual position vector of the target can be expressed as the predicted position vector plus the position error vector, i.e., ***r***_1_ = ***r***_1*o*_ + ***e***_1_, ***r***_2_ = ***r***_2*o*_ + ***e***_2_.

Let *P*_*c*_ denote the collision probability and the coordinate transformation matrix of the navigation and carrier coordinate systems is known. The distance between two targets is the relative position vector modulo *ρ* = |***ρ***| = |***r***_1_-***r***_2_|. The collision probability is expressed as the probability that the distance between two targets is less than the sum of equivalent radii, so the collision probability *P*_*c*_ can be expressed as *P*_*c*_ = *P* {*ρ* ≺ *R* = *R*_1_ + *R*_2_}. Let the starting moment be *t*_0_ = 0, then the relative position vector between the two targets at the moment *t* is:

$$\rho (t\text{)=}r_{1}\left(t\right)-r_{2}\left(t\right)=r_{1}+v_{1}t-r_{2}-v_{2}t=\rho +v_{r}t$$ (8)

Where: ***v***_*r*_ is the relative velocity vector. The square of the distance between the two targets is ***ρ***^2^(*t*) = ***ρ***(*t*) ∙ ***ρ***(*t*), find the derivative concerning time and let the derivative be zero as follows:

$$\begin{array}{l} \frac{d}{dt}\left[\rho^{2}\left(t\right)\right]=\frac{d}{dt}\left[\left(\rho +v_{r}t\right)\cdot \left(\rho +v_{r}t\right)\right]\\ =2\rho \cdot v_{r}+2v_{r}\cdot v_{r}t=0 \end{array}$$ (9)

From Eq (9), the minimum distance between the two targets, i.e., the time to reach the Closest Point of Approach (CPA), is:

$$t_{cpa}=-\frac{\rho \cdot v_{r}}{v_{r}\cdot v_{r}}$$ (10)

Then, the relative position vector of the two objects is:

$$\rho \left(t_{cpa}\right)=\rho +v_{r}t_{cpa}=\rho +\left(-\frac{\rho \cdot v_{r}}{v_{r}\cdot v_{r}}\right)v_{r}$$ (11)

Further:

$$\rho \left(t_{cpa}\right)\cdot v_{r}=\rho \cdot v_{r}-\rho \cdot v_{r}=0$$ (12)

That is, when the distance between two targets is smallest, the dot product of the relative position vector ***ρ***(*t*_*cpa*_) and the relative velocity vector ***v***_*r*_ is 0, which means that the two vectors are perpendicular to each other. In other words, when two objects are closest, they are in the plane perpendicular to the relative velocity vector, which is defined as the Encounter Plane (EP).

**Fig 5:**
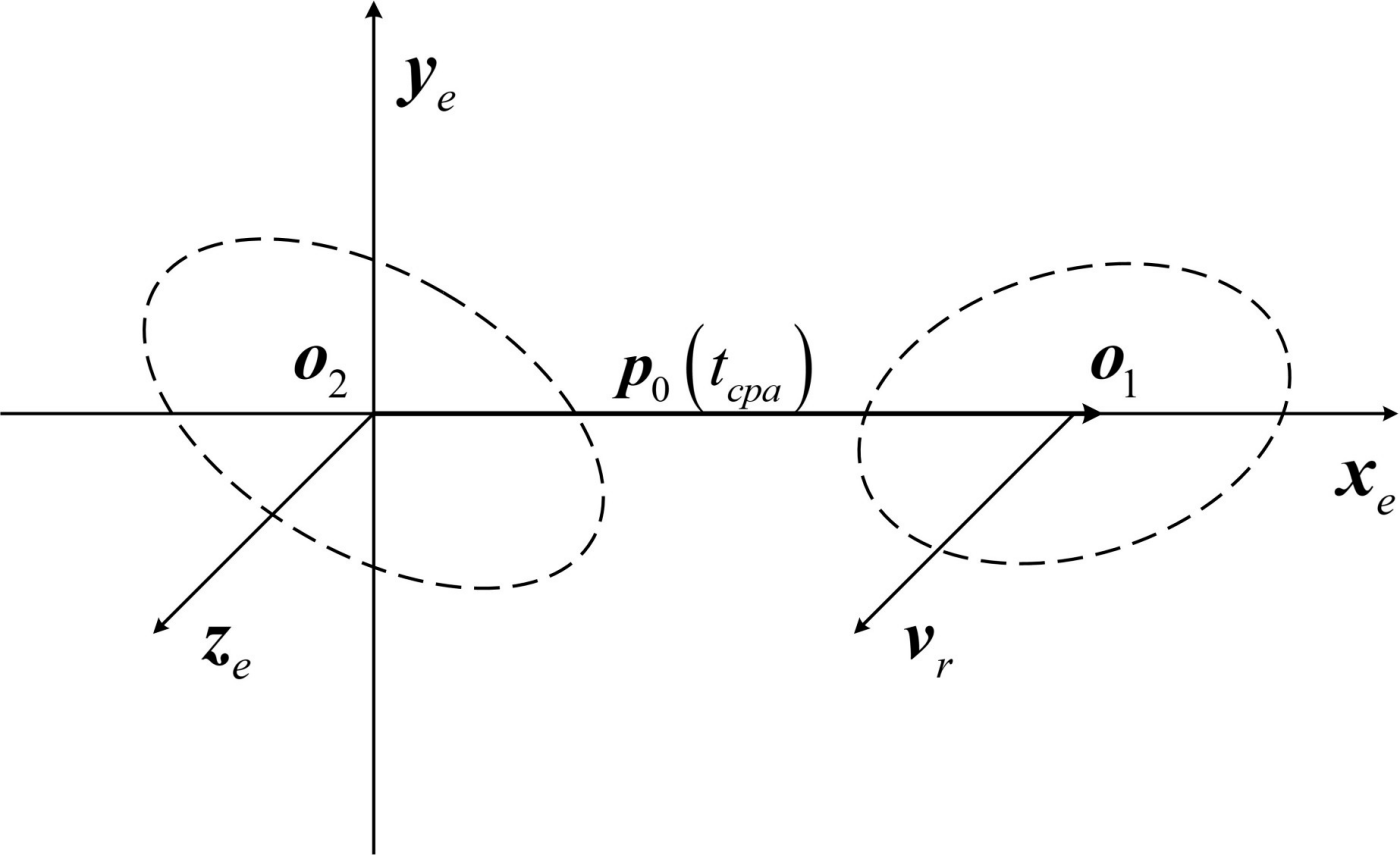
Encounter coordinate system.

The encounter coordinate system *o*–*x*_*e*_*y*_*e*_*z*_*e*_ is defined as shown in Fig 5, where the origin *o*_2_ of the coordinate system is located at the distribution center of the aircraft (target 2), the *x*_*e*_ axis and *y*_*e*_ axis are within the EP and are perpendicular to each other, the *x*_*e*_ axis points to the projection point of the distribution center of the debris (target 1) on the EP, and the ***z***_*e*_ axis point to the direction of the relative velocity vector. According to the definition of the encounter coordinate system, the unit vectors of the three axes of the encounter coordinate system are:

$$i_{e}=\frac{\rho_{0}\left(t_{cpa}\right)}{\left|\rho_{0}\left(t_{cpa}\right)\right|},\;k_{e}=\frac{v_{r}}{\left|v_{r}\right|},\;j_{e}=k_{e}\times i_{e}$$ (13)

Thus, the coordinate transformation matrix ***M***_***e***_ for the navigation coordinate system to the encounter coordinate system:

$$M_{e}={\left[i_{e}\;j_{e}\;k_{e}\right]}^{T}$$ (14)

### 3.2. Position error projection

Suppose a vector is represented in the carrier coordinate system, the navigation coordinate system, and the EP coordinate system as ***r***_***b***_, ***r***_***n***_, ***r***_***e***_, respectively, then the transformation relation is:

$$r_{e}=M_{e}r_{n}=M_{e}R_{b}^{n}r_{b}$$ (15)

The resulting coordinate transformation matrix from the carrier coordinate system to the encounter coordinate system is $M_{e1}=M_{e}R_{b}^{n}$. Let ***X***_***r***_ denote the random vector in the carrier coordinate system, which is represented in the EP coordinate system ***X***_***re***_, and the transformation relationship between them can be expressed as ***X***_*re*_ = ***M***_*e*1_***X***_***r***_. Then the variance matrix ***X***_***re***_ in the encounter coordinate system is:

$$Var\left(X_{re}\right)=M_{e1}Var\left(X_{r}\right)M_{e1}{}^{T}$$ (16)

**Fig 6:**
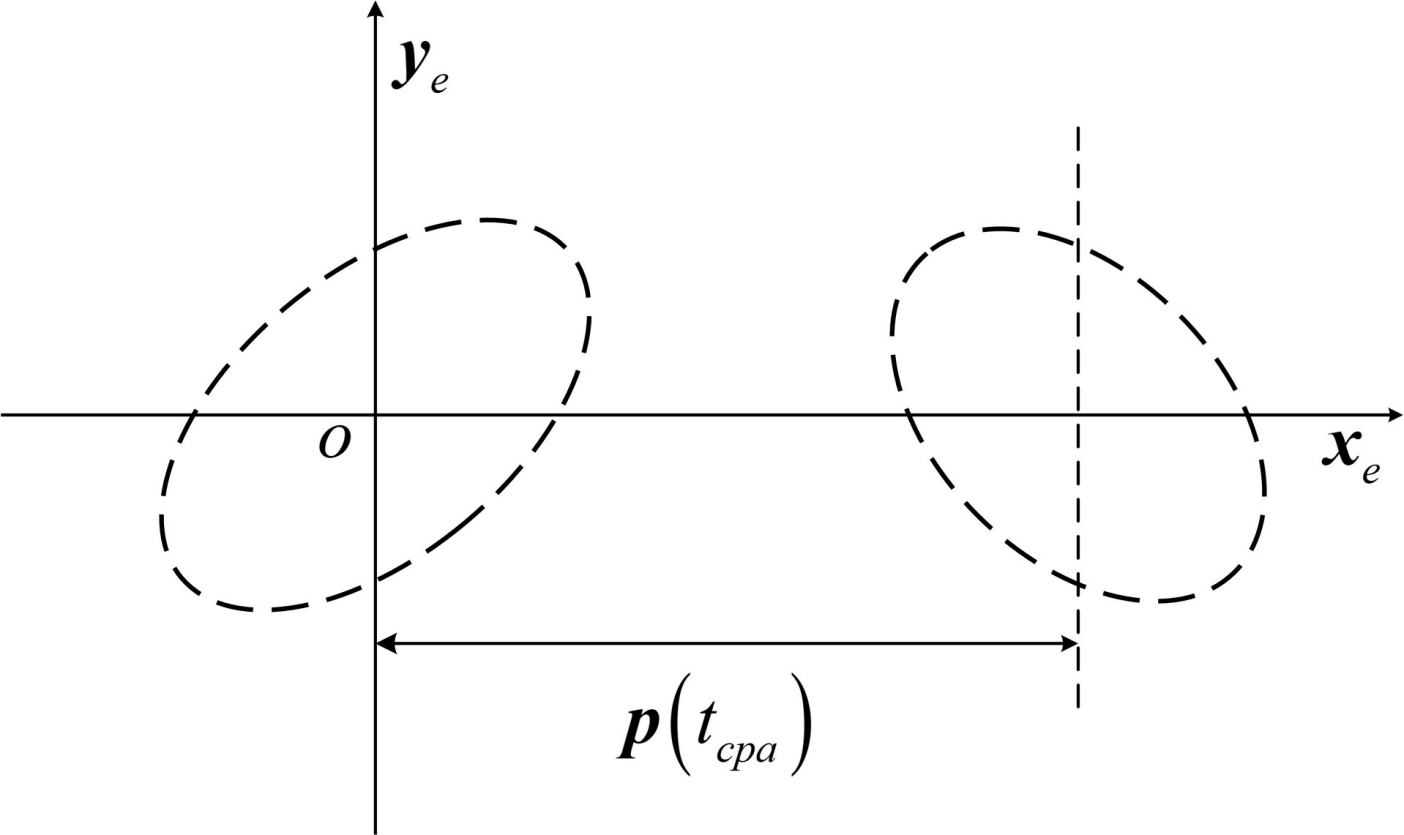
Projected onto the encounter plane.

**Fig 7:**
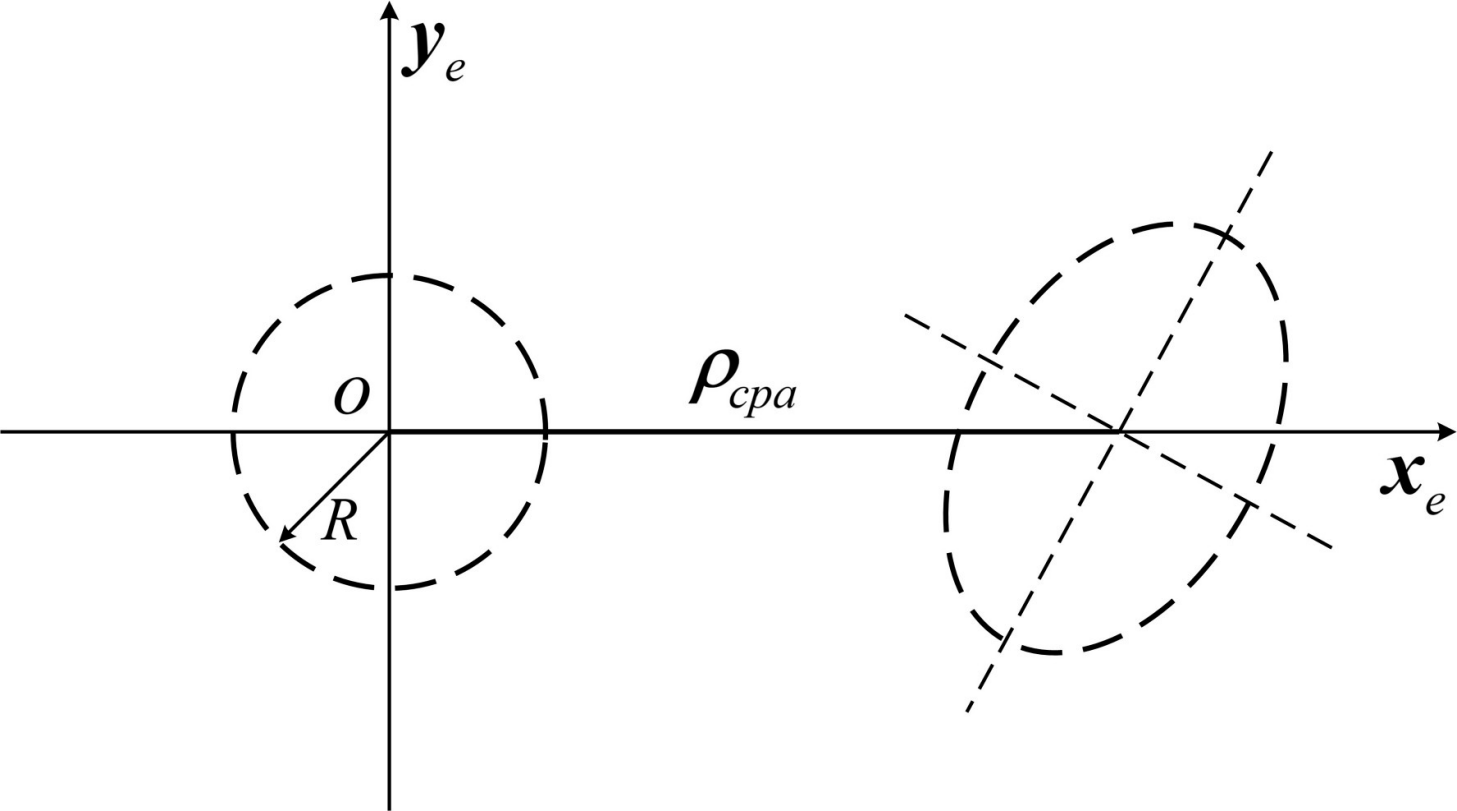
Joint circle domain and joint error ellipse.

This converts the position error covariance matrix represented in the carrier coordinate system to the position error covariance matrix represented in the encounter coordinate system, and by eliminating the terms associated with ***z***_*e*_ in this covariance array, the position error is projected onto the encounter plane, thus reducing the three-dimensional complex problem to a two-dimensional one, as shown in Fig 6:

The position vectors ***X***_1_ and ***X***_2_ of two targets in the encounter plane obey a two-dimensional normal distribution and are independent of each other, then the relative position vector ***X*** = ***X***_1_ –***X***_2_ is also a normal random vector. The collision probability of two targets is the probability that the relative position vectors fall into the circle with *R* = *R*_1_ + *R*_2_ as the radius. Therefore, the joint circular domain is constructed with the origin *o*_2_ as the center and *R* as the radius, and the position error ellipses of the two targets are formed into a joint error ellipse at target 1, as shown in Fig 7:

In general, the variance matrix of the relative position vector ***X*** is not diagonal, i.e., the EP coordinate axes are not parallel to the direction of the principal axes of the joint error ellipse, and to simplify the calculation, the coordinate transformation is needed again. To make the principal axes of the joint error ellipse parallel to the coordinate axes, define a computational coordinate system *o*–*x*_*c*_*y*_*c*_, with the origin coinciding with the encounter coordinate system, the *x*_*c*_ axis pointing in the direction of the short axis of the joint error ellipse in the encounter plane, and *y*_*c*_ pointing in the direction of the long axis. Assuming that an angle of rotation *θ* is required, in the encounter co-ordinate system, let the variance array ***X*** be

$$Var\left(X\right)=\left[\begin{array}{cc} \sigma_{x}^{2} & \kappa \sigma_{x}\sigma_{y}\\ \kappa \sigma_{x}\sigma_{y} & \sigma_{y}^{2} \end{array}\right]$$ (17)

where: *κ* denotes the correlation coefficient. In the computational coordinate system *o*–*x*_*c*_*y*_*c*_, the variance matrix of the random vector ***X*** is

$$Var{\left(X\right)}_{c}=\left[\begin{array}{cc} \sigma_{x}^{'2} & 0\\ 0 & \sigma_{y}^{'2} \end{array}\right]$$ (18)

Based on the relationship between the variance array under coordinate transformation, it follows that

$$Var{\left(X\right)}_{c}=M\left(\theta \right)Var\left(X\right)M^{T}\left(\theta \right)$$ (19)

Where: ***M***(*θ*) is the coordinate transfer matrix from the encounter coordinate system to the calculated coordinate system.

$$M\left(\theta \right)=\left[\begin{array}{cc} \text{cos}\;\theta & \text{sin}\;\theta \\ \text{sin}\;\theta & \text{cos}\;\theta \end{array}\right]$$ (20)

and,

$$Var{\left(X\right)}_{c}=\left[\begin{array}{cc} \sigma_{x}^{2}{\text{cos}}^{2}\;\theta +\kappa \sigma_{x}\sigma_{y}\text{sin}\;2\theta +\sigma_{y}^{2}{\text{sin}}^{2}\;\theta & \frac{\sigma_{y}^{2}-\sigma_{x}^{2}}{2}\text{sin}\;2\theta +\kappa \sigma_{x}\sigma_{y}\text{cos}\;2\theta \\ \frac{\sigma_{y}^{2}-\sigma_{x}^{2}}{2}\text{sin}\;2\theta +\kappa \sigma_{x}\sigma_{y}\text{cos}\;2\theta & \sigma_{x}^{2}{\text{cos}\;}^{2}\;\theta +\kappa \sigma_{x}\sigma_{y}\text{sin}\;2\theta +\sigma_{y}^{2}{\text{cos}}^{2}\;\theta \end{array}\right]$$ (21)

Since *Var*(***X***)_c_ is a diagonal array, it follows that

$$\frac{\sigma_{y}^{2}-\sigma_{x}^{2}}{2}\text{sin}\;2\theta +\kappa \sigma_{x}\sigma_{y}\text{cos}\;2\theta =0$$ (22)

For the axis of rotation *x*_*c*_ to point in the direction of the short semi-axis of the error ellipse, there should be $\sigma_{x}^{'2}<\sigma_{y}^{'2}$, i.e.

$$\frac{\sigma_{y}^{2}-\sigma_{x}^{2}}{2}\text{cos}\;2\theta +\kappa \sigma_{x}\sigma_{y}\text{sin}\;2\theta >0$$ (23)

The angle of rotation *θ* should therefore satisfy the condition that

$$\text{tan}\;2\theta =\frac{-2\kappa \sigma_{x}\sigma_{y}}{\sigma_{y}{}^{2}-\sigma_{x}{}^{2}},\text{tan}\;2\theta <\frac{\sigma_{y}{}^{2}-\sigma_{x}{}^{2}}{2\kappa \sigma_{x}\sigma_{y}}$$ (24)

**Fig 8:**
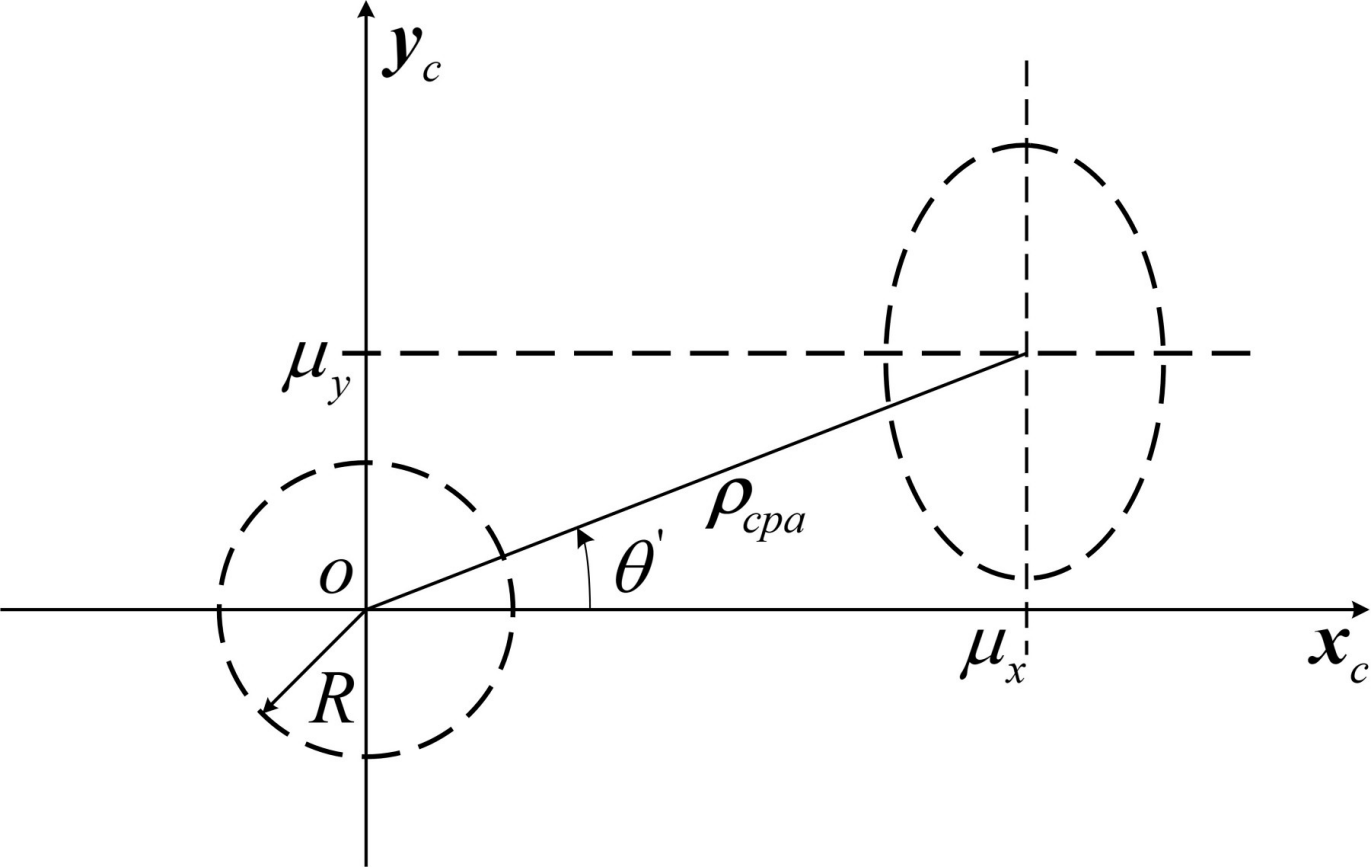
Integral calculation coordinate system.

The distribution center should rotate in the computational coordinate system by an amplitude of *θ*’ = -*θ*, as shown in Fig 8, at which point the parameters of the error distribution in the computational coordinate system are

$$\mu_{x}=\rho_{cap}\;\text{cos}\left(\theta '\right)$$ (25)

$$\mu_{y}=\rho_{cap}\;\text{sin}\left(\theta '\right)$$ (26)

$$\sigma_{x}^{'2}=\sigma_{x}^{2}\;{\text{cos}}^{2}\theta +\kappa \sigma_{x}\sigma_{y}\;\text{sin}2\theta +\sigma_{y}^{2}\;{\text{sin}}^{2}\theta$$ (27)

$$\sigma_{y}^{'2}=\sigma_{x}^{2}\;{\text{sin}\;}^{2}\theta -\kappa \sigma_{x}\sigma_{y}\;\text{sin}\;2\theta +\sigma_{y}^{2}\;{\text{cos}\;}^{2}\theta$$ (28)

The two-dimensional normal distribution probability density function (PDF) is:

$$f\left(x,y\right)=\frac{1}{2\pi \sigma_{x}^{'}\sigma_{y}^{'}}\text{exp}\left[-\frac{1}{2}\left(\frac{{\left(x-\mu_{x}\right)}^{2}}{\sigma_{x}^{'2}}+\frac{{\left(y-\mu_{y}\right)}^{2}}{\sigma_{y}^{'2}}\right)\right]$$ (29)

The collision probability *P*_*c*_ is the integral of the PDF in the joint circular domain:

$$P_{c}=\iint_{x^{2}+y^{2}\leq R^{2}}f\left(x,y\right)dxdy$$ (30)

In this way, the problem of calculating probability is transformed into the problem of integrating the probability density function in the circle domain.

## 4. Example analysis

Statistical data show that the aircraft in cruise is in a relatively stable state with typical motion characteristics, and its motion is considered to satisfy a typical linear growth pattern without additional assumptions, where the typical airspeed of a commercial transport aircraft is 0.25 km/s. The motion of the vehicle before the disintegration accident is usually known from radar observations and flight trajectory equations, and the international common spacecraft The disintegration altitude is 78 km, and the yaw angle *ѱ*_*0*_ = 0, pitch angle *θ*_*0*_ = -1, initial velocity *v*0 = 7.1km/s, and initial longitude and latitude of the suborbital vehicle before disintegration are assumed to be *γ*_*0*_ = 157°W and *φ*_*0*_ = 20°N, respectively. The aircraft model takes the Boeing 737 as an example, and the aircraft’s equivalent radius can be set to 30 m. In this modeling, only the individual debris form is considered, and the suborbital debris equivalent radius is set to 0.2 m based on the debris statistics of the Columbia disintegration accident, and the joint equivalent radius is R = 30.2 m.

Through the above calculation, when the debris of the suborbital disintegration accident falls to the civil aviation altitude, the state information of the aircraft and some debris at the reference moment is given in combination with the aircraft trajectory prediction model, and the collision probability, the closest moment and the closest distance between the aircraft and each debris are calculated respectively, as shown in Table 1.

**Table 1:** Analysis table of the collision probability between the aircraft and different debris at the reference moment.

Reference moment		Airplane	01 debris	02 debris	03 debris	04 debris
***Location*/*km***	X Y Z v_*x*_	331.70 -22.64 10.34 9.38×10^−3^	331.60 -22.58 10.34 1.80×10^−3^	331.45 -22.59 10.34 1.80×10^−3^	331.38 -22.57 10.34 2.0×10^−3^	332.19 -22.44 10.34 2.0×10^−3^
***Speed/(km***·***s***^**-1**^***)***	v_*y*_ v_*z*_	0.25 0	5.10×10^−3^ -17.50×10^−3^	3.65×10^−3^ -17.1×10^−3^	3.95×10^−3^ -16.5×10^−3^	3.65×10^−3^ -16.5×10^−3^
***Closest moment*/*s*** ***Closest distance/km***	t_*cpa*_ p_0*cpa*_		0.2312 0.1019	0.1079 0.2514	0.2443 0.3200	0.8675 0.4840
***Collision probability***	P_*c*_		3.6×10^−3^	2.45×10^−4^	2.05×10^−5^	6.52×10^−7^

### 4.1. Debris position error distribution

It is assumed that the error of each debris position is spherically distributed, that is:

$$Var\left(r_{1}\right)=\left[\begin{array}{ccc} \sigma_{x}{}^{2} & 0 & 0\\ 0 & \sigma_{y}{}^{2} & 0\\ 0 & 0 & \sigma_{z}{}^{2} \end{array}\right]=\left[\begin{array}{ccc} 0.01 & 0 & 0\\ 0 & 0.01 & 0\\ 0 & 0 & 0.01 \end{array}\right]$$ (31)

**Fig 9:**
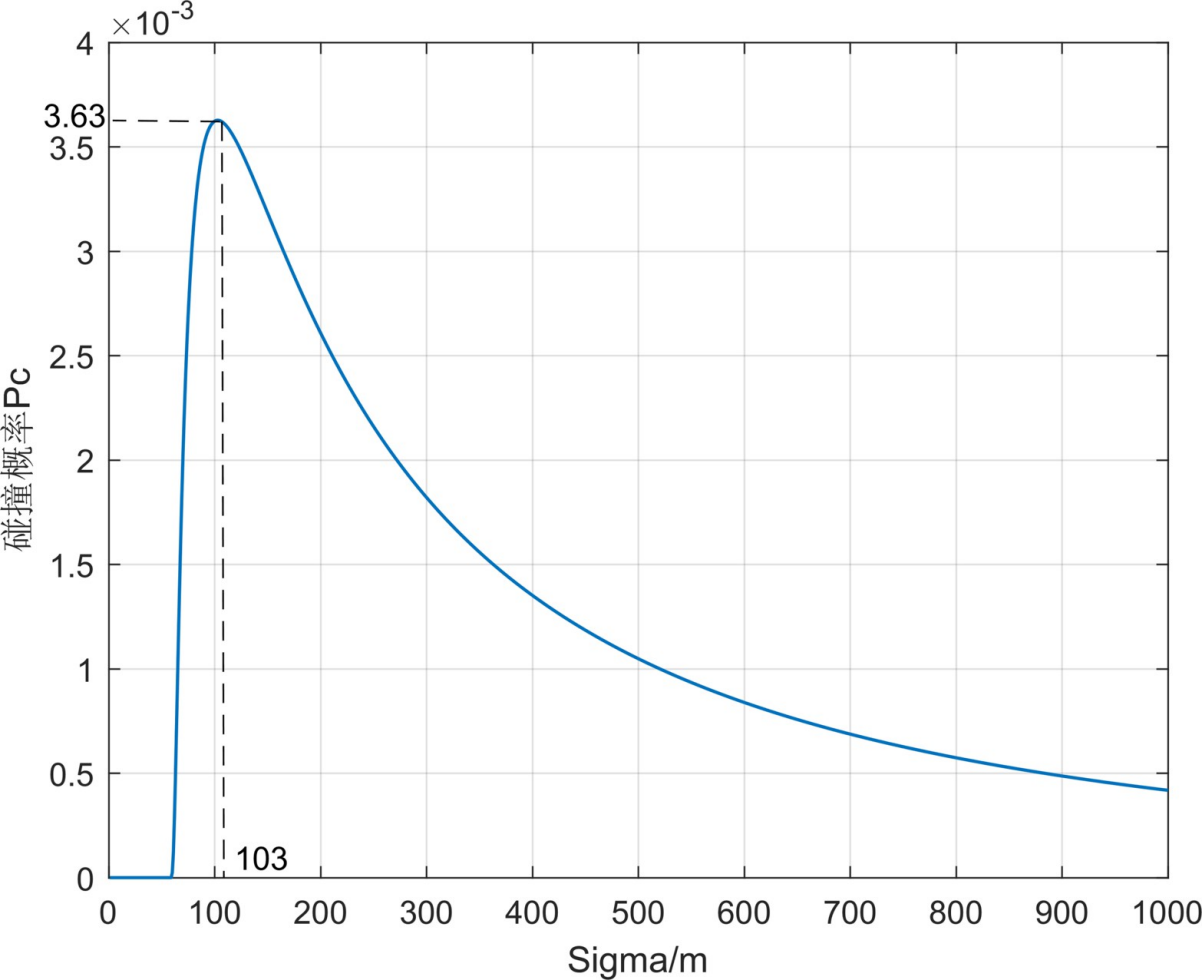
*P*_*c*_ values with *σ*_*x*_ changing when *σ*_*x*_: *σ*_*y*_: *σ*_*z*_ = 1:1:1.

**Fig 10:**
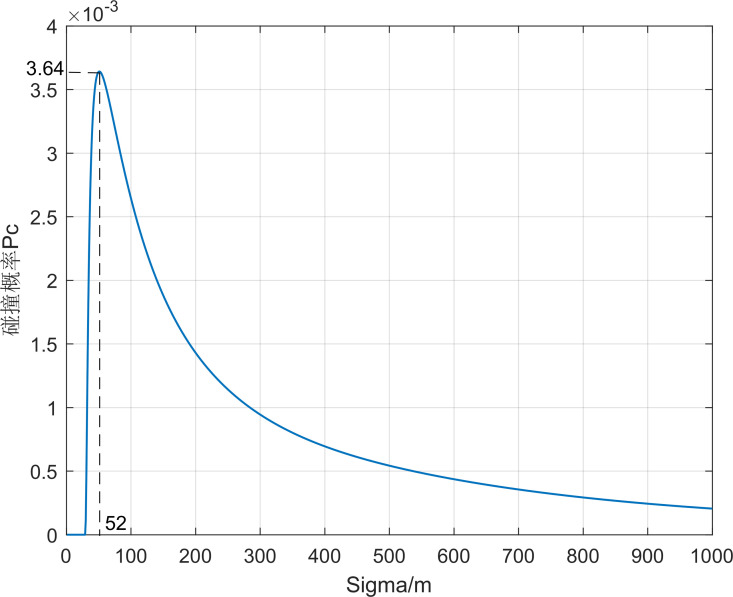
*P*_*c*_ values with *σ*_*x*_ changing when *σ*_*x*_: *σ*_*y*_: *σ*_*z*_ = 1:1:2.

**Fig 11:**
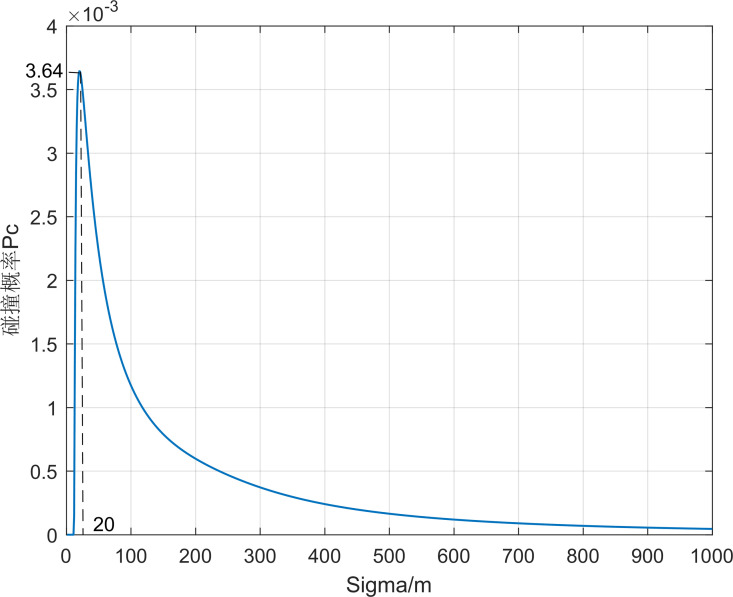
*P*_*c*_ values with *σ*_*x*_ changing when *σ*_*x*_: *σ*_*y*_: *σ*_*z*_ = 1:1:5.

Take the first set of simulation data and calculate the collision probability between the aircraft and debris 01, as shown in Fig 9. The collision probability *P*_*c*_ increases first with the increase of position error and starts to decrease after *σ*_*x*_ = 103 m reaching a great value *P*_*c*max_. When the position error is small, as long as the aircraft and the debris are not in the intersecting path, the probability of collision is small. As the position error increases, the probability of collision increases, and after reaching the maximum value, as the error range continues to increase, the probability of collision is small even if the distance between the aircraft and the debris is small. In addition, in the actual situation the debris position error is larger in the direction of the ***z*** axis, therefore, considering the position error distribution *σ*_*x*_: *σ*_*y*_: *σ*_*z*_ increases from 1:1:1 to 1:1:5, as seen in Figs 10 and 11, the collision probability extreme value point keeps shrinking.

### 4.2. Airborne risk target collision warning

The international general spacecraft maneuver avoidance probability yellow threshold value is *p*_*c*_ = 10^−5^, the red threshold is *p*_*c*_ = 10^−4^, according to the calculation results in Table 1, it is known that the collision probability between the aircraft and debris 01 and 02 are greater than and equal to the red threshold value, respectively, and the collision probability with debris 03 is in the yellow warning level, there is a possibility of collision and need to take avoidance maneuvers in advance. In addition, the collision probability between the aircraft and debris 04 is much smaller than the yellow warning value and will be a safe rendezvous event. In summary, to avoid the safety impact of suborbital disintegration on the civil airliner, the collision probability value between the aircraft and any debris in the debris group should be less than the yellow warning value, and the collision probability between the aircraft and the debris should be reduced to below 10^−7^ after the avoidance measures are taken.

### 4.3. Fragmentation prediction calculation efficiency

**Fig 12:**
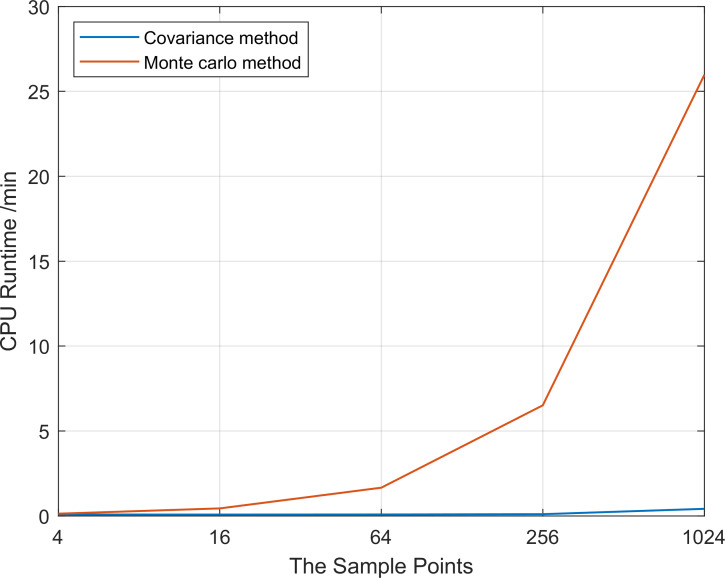
Comparison of CPU runtime.

The covariance propagation method used in this paper has a more significant advantage in running time compared to the traditional Monte Carlo algorithm while increasing the sampling points, as shown in Fig 12, which will facilitate the real-time response work of ATMs.

## 5. Conclusion

In this paper, the covariance propagation algorithm is used to model and analyze the debris drop points generated by suborbital vehicle disintegration accidents. Compared with the traditional Monte Carlo algorithm, the computational efficiency of this algorithm is significantly improved. Combined with the aircraft trajectory prediction model, the position and velocity information of the aircraft and each suborbital debris at the closest moment in the same altitude level are predicted in advance, and the position error covariance matrix of the aircraft and suborbital debris are combined by the above method. After linear transformation and coordinate conversion, the problem of collision probability calculation is converted into the problem of integrating the probability density function of two-dimensional normal distribution in a circular domain. Concerning the international common standard for spacecraft avoidance maneuvers, the collision warning of airborne risk targets is carried out, and an important foundation for the construction of a safe and efficient decision support system for flight avoidance is laid.

## References

[1] Gong Y K, Qin T, Wei W, Mou Y. Analysis of international commercial space market and policy[J]. Aerospace China, 2020, 20(4): 39–48. doi: 10.3969/j.issn.1671-0940.2019.04.005

[2] Zahari A R, Romli F I. Analysis of suborbital flight operation using PESTLE[J]. Journal of Atmospheric and Solar-Terrestrial Physics, 2019, 192: 104901. doi: 10.1016/j.jastp.2018.08.006

[3] AST F. The annual compendium of commercial space transportation: 2018[J]. Federal Aviation Administration, 2018.

[4] Paul Fitzgerald P. Inner space: ICAO’s new frontier[J]. Journal of Air Law and Commerce, 2014, 78(4): 3–34.

[5] Rui-feng Hu, Zi-zheng Gong, Zi-niu Wu. Engineering methods for reentry prediction of uncontrolled spacecraft and space debris: the state of the art (in Chinese) [J]. Spacecraft Environment Engineering, 2014,31(05):548–557.

[6] Wilde P, Draper C. Aircraft protection standards and implementation guidelines for range safety[C]//48th AIAA Aerospace Sciences Meeting Including the New Horizons Forum and Aerospace Exposition. 2010: 1542. doi: 10.2514/6.2010-1542

[7] Murray D. The FAA’s Current Approach to integrating commercial space operations into the national airspace system[J]. Federal Aviation Administration, 2013.

[8] Liang Y, Chen W, Feng S, et al. Risk modelling and probability analysis of civil aircraft operation in suborbital disintegration accident[C]//International Conference on Sensors and Instruments (ICSI 2021). International Society for Optics and Photonics, 2021, 11887: 118870H.

[9] Morlang F, Ferrand J, Seker R. Why a future commercial spacecraft must be able to SWIM[J]. Journal of Space Safety Engineering, 2017, 4(1): 5–8. DOI: 10.1016/j.jsse.2017.03.003

[10] Hays C, Chu D, Llanos P. A statistical approach for commercial space vehicle integration into the national airspace system[J]. 2019.

[11] Luchkova T, Kaltenhaeuser S, Morlang F. Air traffic impact analysis design for a suborbital point-to-point passenger transport concept[J]. 2016.

[12] Sarconi M. A prototype system for simulating the risks of sub-orbital space flight for commercial aviation[R]. Glasgow, UK: Level 4 Project, School of Computing Science University of Glasgow, 2013.

[13] Johnson C W. Using the ‘Internet of Things’ to Support Dynamic Risk Assessment in Future Concepts of Operation for Air Traffic Management[D]. Glasgow: University of Glasgow, 2015.

[14] Reyhanoglu M, Alvarado J. Estimation of debris dispersion due to a space vehicle breakup during reentry[J]. Acta Astronautica, 2013, 86: 211–218. DOI: 10.1016/j.actaastro.2013.01.018

[15] Picone J M, Hedin A E, Drob D P, et al. NRLMSISE-00 empirical model of the atmosphere: Statistical comparisons and scientific issues[J]. Journal of Geophysical Research: Space Physics, 2002, 107(A12): SIA 15-1-SIA 15–16. doi: 10.1029/2002JA009430

[16] Drob D P, Emmert J T, Crowley G, et al. An empirical model of the Earth’s horizontal wind fields: HWM07[J]. Journal of Geophysical Research: Space Physics, 2008, 113(A12).

[17] Paielli R A, Erzberger H. Conflict probability estimation for free flight[J]. Journal of Guidance, Control, and Dynamics, 1997, 20(3): 588–596. doi: 10.2514/2.4081

[18] Prandini M, Hu J, Lygeros J, et al. A probabilistic approach to aircraft conflict detection[J]. IEEE Transactions on intelligent transportation systems, 2000, 1(4): 199–220.

[19] Bai Xian-zong. Research on collision probability in space objects collision detection (in Chinese) [D]. National University of Defense Technology, 2008.

